# Role of Intensified Lung Physiotherapy Bundle on the Occurrence of Pneumonia After Cardiac Surgery

**DOI:** 10.3389/fmed.2022.844094

**Published:** 2022-02-23

**Authors:** Wei Cheng, Jianwei Chen, Jianhua Sun, Jiahui Zhang, Dongkai Li, Hao Wang, Zunzhu Li, Na Cui

**Affiliations:** ^1^State Key Laboratory of Complex Severe and Rare Diseases Department of Critical Care Medicine,Peking Union Medical College, Peking Union Medical College Hospital, Chinese Academy of Medical Science, Beijing, China; ^2^Department of Critical Care Medicine, Beijing Jishuitan Hospital, Beijing, China

**Keywords:** cardiac surgery, lung physiotherapy bundle, postoperative pneumonia, mechanical ventilation time, in-hospital mortality

## Abstract

**Objective:**

The role of intensified lung physiotherapy bundle after cardiac surgery was investigated.

**Methods:**

A before- and after-surgery comparison was conducted between the study from January 1, 2018 to December 31, 2019 (control group), when traditional lung physiotherapy bundle was used, and from January 1, 2020 to May 1, 2021 (study group), when the intensified bundle was used. The baseline data, clinical features, incidence of postoperative pneumonia, and prognoses of all the enrolled cardiac surgery patients were analyzed.

**Results:**

In accordance with the study criteria, 358 patients were enrolled. The incidence rate of postoperative pneumonia was significantly lower in the study group than in the control group (14.2 vs. 22.7%, *P* = 0.037), as was in-hospital mortality (1.5 vs. 5.2%, *P* = 0.043). Patients receiving the intensified lung physiotherapy bundle had much shorter mechanical ventilation time (92 vs. 144 h, *P* < 0.0001), much shorter intensive care unit (ICU) stay (5 vs. 7 days, *P* < 0.001), and much shorter hospital stay (17 vs. 18.5 days, *P* = 0.022). The intensified lung physiotherapy bundle was an independent protective factor enabling the reduced occurrence of pneumonia (*P* = 0.007). On univariate analysis, this bundle significantly improved in-hospital mortality (*P* = 0.043).

**Conclusions:**

Our intensified lung physiotherapy bundle potentially reduces the rate of postoperative pneumonia after cardiac surgery. This bundle might also be adopted as a suitable reference guide for the prevention of other postoperative pulmonary complications.

## Introduction

Pneumonia is a common cause of respiratory failure after cardiac surgery, while cardiopulmonary bypass can activate the inflammatory response, cause diffuse endothelial cell injury, and lead to non-cardiac pulmonary oedema and bilateral lung infiltration. Given the depressed cardiac function and high risk of bleeding following cardiac surgery, patients are often kept on deep sedation and analgesia in a supine position without regularly being turned over and other lung physiotherapy strategies, making sputum drainage more difficult and increasing the probability of postoperative pneumonia. The incidence of pneumonia after cardiac surgery has been reported to be as high as 20%, with the mortality rate of patients with postoperative pneumonia as high as 30% (1–3).

Lung physiotherapy can be used as an adjunctive measure in the treatment of respiratory failure in the intensive care unit (ICU) by clearing secretions, increasing pulmonary volume, and re-inflating atelectatic lungs (4, 5). Lung physiotherapy has also been proved to reduce the incidence of ventilator-associated pneumonia, the duration of mechanical ventilation, and ICU stay in patients with severe pneumonia and to reduce the incidence of pulmonary complications after cardiac surgery (6). However, as of now, there is no standard detailed lung physiotherapy protocol for clinical application and no dynamic adjustment according to the departmental situation.

Our department has always attached great importance to lung physiotherapy. In 2018, based on the actual situation in our department, we conceived and formulated a modified set of lung physiotherapy measures more suitable for patients with severe pneumonia, which we called the intensified lung physiotherapy bundle. The application of this bundle in patients with sepsis confirmed their favorable effect on mortality (7, 8). Therefore, from January 1, 2020, we introduced the intensified lung physiotherapy bundle for all cardiac surgery patients admitted to our ICU. After more than 1 year of clinical practice, we explored to what extent this bundle could reduce the incidence of pneumonia after cardiac surgery and improve the prognosis of the affected patients.

## Methods

### Study Design

This controlled before-and-after study enrolled cardiac surgery patients admitted to our ICU at Peking Union Medical College Hospital (PUMCH). The patients in the control group were enrolled from January 1, 2018 to December 31, 2019, when a traditional lung physiotherapy bundle was used, while patients in the study group were enrolled from January 1, 2020 to May 1, 2021, when the intensified lung physiotherapy bundle was applied for all cardiac surgery patients. This study was approved by the Institutional Review Board of PUMCH (approval number: JS-1170). Consent forms were obtained from the patients' next of kin at ICU admission. The study was registered at chictr.org.cn (identifier: ChiCTR1900025850).

**Figure 1:**
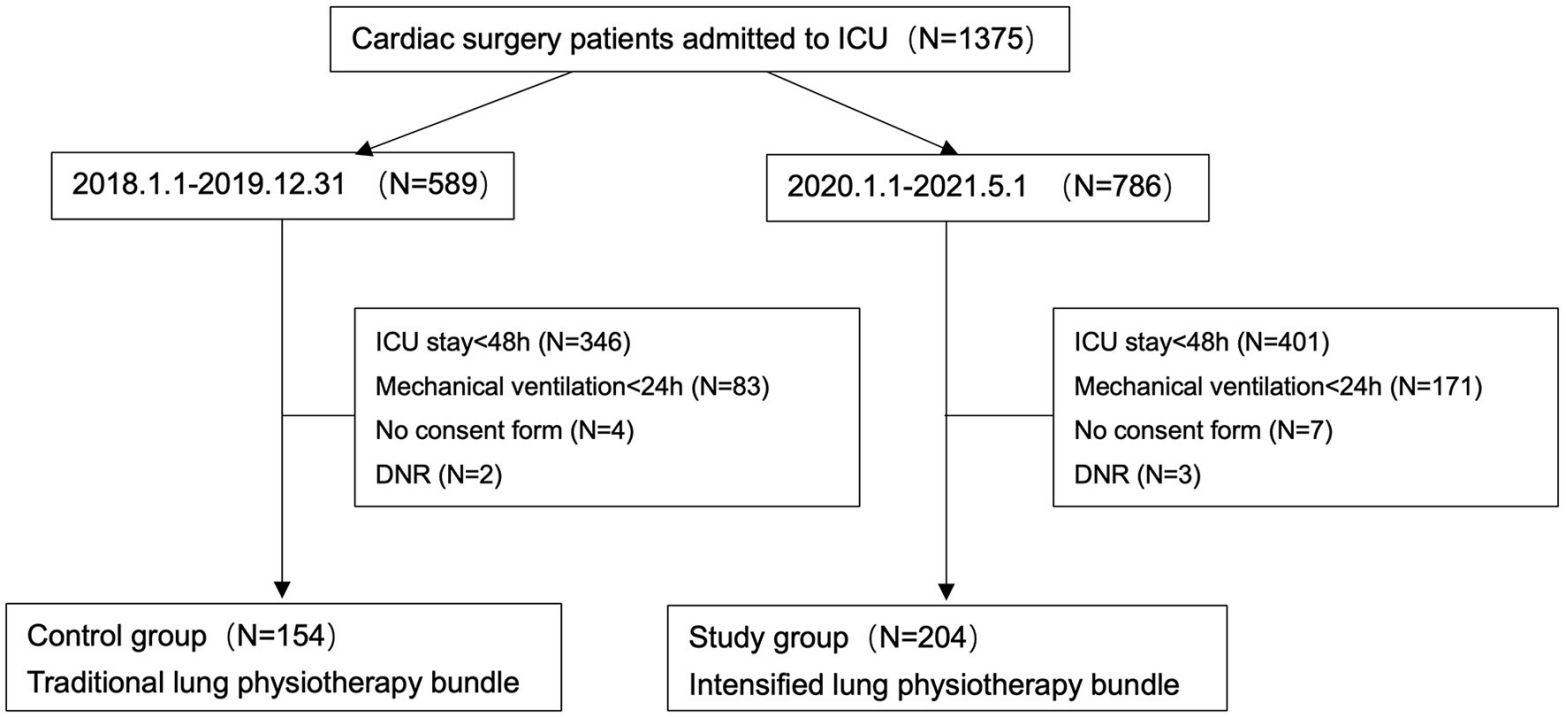
Traditional and intensified lung physiotherapy bundle and treatment flowchart.

The traditional and intensified lung physiotherapy bundle was performed as described in previously published literature (7). The traditional lung physiotherapy included (1) bed head elevation at 30–45°; (2) daily sedation depth assessment, adjusting sedative drugs to maintain the Richmond Agitation sedation score (RASS) −2–−1; (3) routine mouth care with chlorhexidine three times a day; (4) sputum aspiration every 2 h or as needed, use of subglottic suction device; (5) conduction of regular auscultation. Compared with the traditional one, the intensified bundle has several modifications: (1) delirium was assessed every 6 h by the Confusion Assessment Method for ICU (CAM-ICU) at the same time as the daily assessment of sedation depth; (2) the frequency of oral care was determined by the modified Beck Oral Assessment Scale score (BOAS) and mucosal-plaque score, which was performed at least every 12 h; (3) enhanced airway drainage with a vibratory sputum extractor every 4 h for 20–30 min. Coughing intensity was assessed every 6 h and the degrees of temperature and humidity were adjusted according to the sputum properties after sputum aspiration to achieve a sputum viscosity of grade II; (4) all the patients with unsatisfied lung assessment (including auscultation, lung ultrasound, and chest X-ray) received lung recruitment maneuver and high lateral position (left side or right side: >90° and the position was changed every 2 h); (5) early activity as soon as possible: sitting up and active motion on bed when vasoactive-inotropic score (VIS) <80, and bedside activity (sitting on wheelchair or assisted walking) when VIS <50 (9). The detailed lung physiotherapy bundle and treatment flowchart are shown in Figure 1.

Patients who met all three of the following criteria were included: (1) post-cardiac surgery; (2) The role of lung physiotherapy would not be reflected if there was too short a time, and the stay in ICU should be longer than 48 h; (3) Continuing mechanical ventilation for at least 24 h for any reasons after surgery. The exclusion criteria were: (1) refusal to sign consent form; (2) patients with pregnancy or lactation; (3) do not resuscitate or admission for palliative care only.

The criteria for the diagnosis of postoperative pneumonia were as follows (3):

(1) At least two of the following symptoms or signs:

- temperature >38 or <36°C;
- white blood cell (WBC) count >12,000 or <4,000 cells mm^−3^;
- newly emerged purulent tracheal secretions;
- decrease in oxygenation: which was defined as PaO2/FiO2 <300 mmHg, and at least two intensivists carefully evaluated the patients to exclude those caused by heart failure or volume overload.

(2) New-onset or progressive pulmonary infiltrates caused by infection.

(3) Organisms isolated from sputum obtained by transtracheal aspiration.

Lower respiratory tract sputum was obtained after ICU admission and sent immediately to the PUMCH Clinical Microbiology Laboratory. Sputum was obtained using non-invasive sampling and was cultured semi-quantitatively. Endotracheal aspirates with >25 WBCs and <10 epithelial cells per high-power field on Gram staining were required for culture.

### Data Collection

Age, sex, underlying diseases, vital signs, ventilator parameters (positive end-expiratory pressure [PEEP], tidal volume, the fraction of inspired oxygen [FiO_2_]), blood gas analysis, and laboratory evaluations were collected. Life-sustaining treatments included vasopressors, cardiotonic agents, continuous renal replacement therapy (CRRT), intra-aortic balloon pump (IABP), and extracorporeal membrane oxygenation (ECMO). The Sequential Organ Failure Assessment (SOFA) score, Acute Physiology and Chronic Health Evaluation II (APACHE II) score, and Clinical Pulmonary Infection Score (CPIS) were calculated on the first ICU Day. The outcomes included postoperative pneumonia, duration of mechanical ventilation, duration of ICU stay, duration of hospital stay, and in-hospital mortality.

### Statistical Analysis

First, all data were tested to define the normality. Normally distributed data were expressed as mean ± SD. Non-normally distributed data were expressed as median and interquartile range and were analyzed using the Mann–Whitney U test. Categorical variables were presented as numbers and percentages and analyzed using the Chi-squared test or Fisher's exact test. Variables with a *P-*value of <0.05 were statistically significant and were included in the multivariate regression to determine significantly independent risk factors. The results were analyzed using SPSS Version 23.0 (IBM, Armonk, NY, USA).

## Results

**Figure 2:**
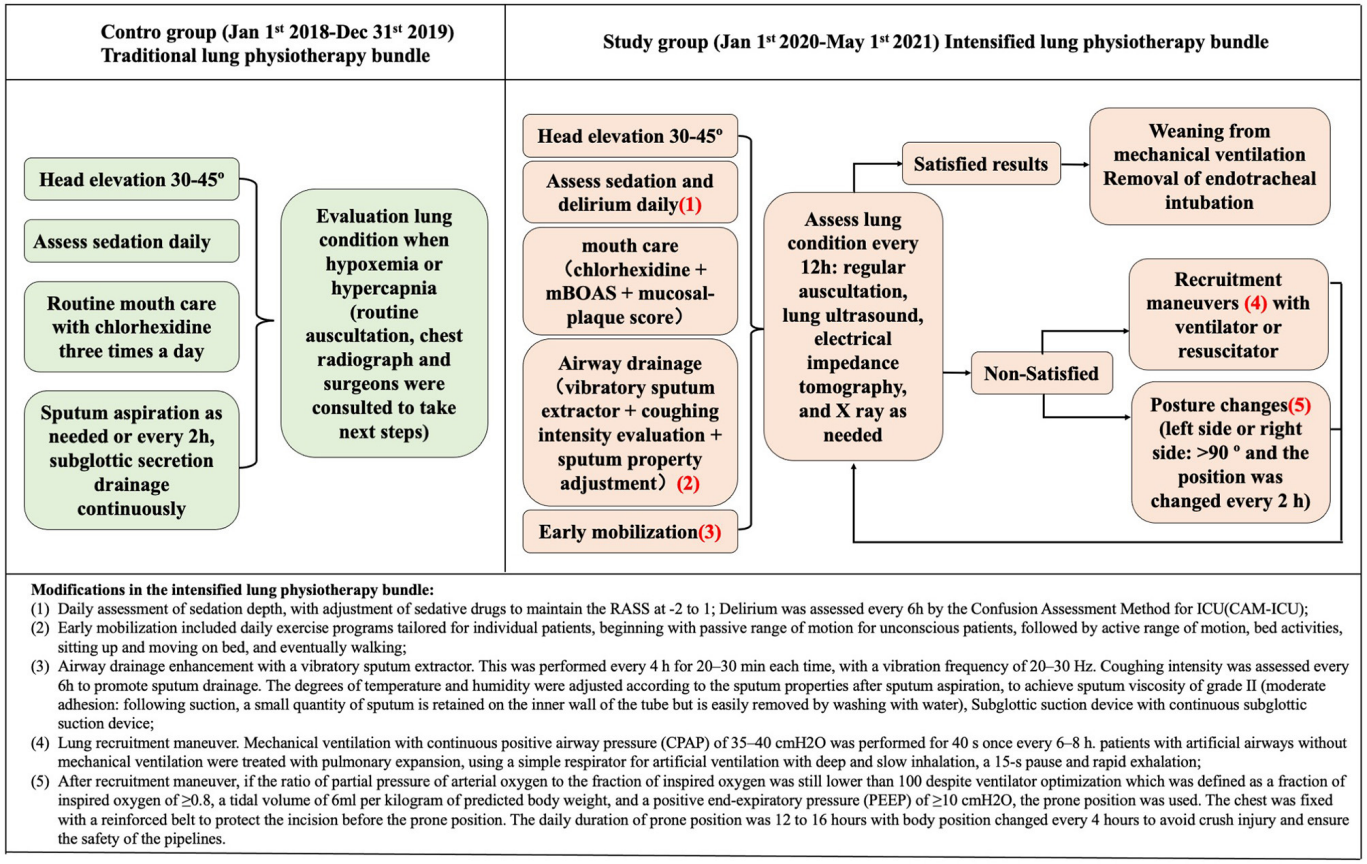
Enrolment flowchart. ICU, intensive care unit; DNR, do not resuscitate.

From January 1, 2018 to May 1, 2021, a total of 1,375 cardiac surgery patients were transferred to the ICU because of high surgical risk. A total of 589 patients were admitted between January 1, 2018 and December 31, 2019, who received traditional lung physiotherapy immediately after admission. A total of 786 patients were admitted from January 1, 2020 to May 1, 2021 when the intensified lung physiotherapy bundle was performed. Since the purpose of the study was to determine the incidence of postoperative pneumonia, patients who underwent extubation within 24 h after surgery or transferred out of ICU within 48 h were excluded. At last, a total of 154 patients (26.1%, 154/589) were enrolled in the control group and 204 patients (26%, 204/786) were enrolled in the study group (Figure 2).

### Baseline Characteristics of Cardiac Surgery Patients

The 358 patients who met the criteria (>48 h in ICU and >24 h on ventilator) were enrolled with a median age of 59 (47–67) years, 66.5% (238 cases) of whom were male. The median APACHE II score was 16. There was no significant difference in age, sex, morbidities, and disease severity between the two groups. Among these patients, 19.8% (71/358) underwent emergency surgery, 15.9% (57 cases) had infective endocarditis, and 10.3% (37 cases) had aortic dissection/aneurysm. The median cardiopulmonary bypass (CPB) time and aortic blocking time were 128.5 and 87 min, respectively (Table 1). As preoperative pulmonary infection had a great impact on the outcome, it was carefully examined. Based on the WBC counts, Chest X-rays, and clinical symptoms and signs, no patients were actually diagnosed with the pulmonary infection before surgery in both groups

**Table 1:** Baseline characteristics of cardiac surgery patients.

	**Total**	**Control (154)**	**Treatment (204)**	***P***
**Age (years)**	59 (47, 67)	59 (47, 67)	59 (47, 67)	0.885
**Sex (male)**	238 (66.5%)	99 (64.3%)	139 (68.1%)	0.445
**Underlying Disease**				
Hypertension	103 (28.8%)	50 (32.5%)	53 (26%)	0.179
Diabetes Miletus	48 (13.4%)	21 (13.6%)	27 (13.2%)	0.912
COPD	28 (7.8%)	12 (7.8%)	16 (7.8%)	0.986
Chronic Kidney Disease	13 (3.6%)	5 (3.2%)	8 (3.9%)	0.735
Chronic Liver Disease	13 (3.6%)	6 (3.9%)	7 (3.4%)	0.816
**APACHE II**	16 (13, 20)	16 (13, 20)	16 (12, 20)	0.858
**SOFA**	10 (8, 12)	11 (7, 13)	10 (8, 12)	0.361
**Operation Type**				0.248
Emergency surgery	71 (19.8%)	30 (19.5%)	41 (20.1%)	
Pericardial surgery	21 (5.9%)	8 (5.2%)	13 (6.4%)	
Dissection/Aneurysm	37 (10.3%)	14 (9.1%)	23 (11.3%)	
Infectious Endocarditis	57 (15.9%)	33 (21.4%)	24 (11.8%)	
Elective CABG/Valve surgery	132 (36.9%)	52 (33.8%)	80 (39.2%)	
Others^*^	39 (10.9%)	16 (10.4%)	23 (11.3%)	
**Cardiopulmonary Bypass Time (min)**	128.5 (96, 173.5)	122 (94.5, 156)	131 (96.5, 176)	0.133
**Aortic Cross Clamp Duration (min)**	87 (56, 119)	80.5 (62.25, 118.5)	91 (54, 119)	0.867

* *Other surgeries include Pulmonary endarterectomy and congenital heart disease surgery*.

*COPD, chronic obstructive pulmonary disease; APACHE II, acute physiology and chronic health evaluation II; SOFA, sequential organ failure assessment; CABG, coronary artery bypass grafting*.

In terms of clinical features, the vital signs at admission were not statistically different. There were also no significant differences in ventilator parameters (PEEP, respiratory rate, tidal volume, and FiO_2_) and use of prophylactic antibiotics (94.2% vs. 89.2%, *P* = 0.100), use of acid-suppressing drugs (26.6% vs. 24.5%, *P* = 0.649) between the two groups. A total of 51.1% of patients used cardiotonic drugs and 82.1% used norepinephrine after surgery, neither of which showed a significant difference between the two groups. There was also no difference in the application of IABP, ECMO, and CRRT. The lactate and cardiac troponin I (cTnI) levels of the study group were statistically higher than those of the control group (2.1 vs. 1.6 mmol L^−1^ [*P* = 0.038] and 6.9 vs. 2.9 μg L^−1^ [*P* < 0.001], respectively). The levels of interleukin (IL)-8 and tumor necrosis factor-α in the study group were significantly lower than those in the control group (91 vs. 114 pg mL^−1^ [*P* = 0.026] and 2 vs. 14.9 pg mL^−1^ [*P* < 0.0001], respectively), whilst the high-sensitivity C-reactive protein level was significantly higher (167.1 vs. 110.3 mg L^−1^, *P* < 0.0001). Nutritional status had an important influence on the occurrence of postoperative pneumonia, but there was no significant difference in the albumin levels between the two groups (33 vs. 33 g/L, *P* = 0.929), and there were no significant differences in other clinical and laboratory parameters between the two groups (Table 2).

**Table 2:** Clinical characteristics of cardiac surgery patients at ICU admission.

	**Total**	**Control group**	**Study group**	***P***
**Vtial signs at admission**				
Heart rate (beats per minute)	104 (92, 113)	103 (92, 111)	104 (93, 115)	0.197
Mean artery pressure (mmHg)	82 (75, 88)	85 (75, 88)	81 (75, 88)	0.178
Temperature (°C)	37.8 (37.3, 38.2)	37.8 (37.3, 38.1)	37.8 (37.3, 38.2)	0.314
Respiratory rate (per minute)	16 (15, 20)	17 (15, 23)	16 (15, 18)	0.01
**Ventilation parameters**				
PEEP (cmH2O)	6 (5, 8)	6 (5, 8)	6 (5.8)	0.347
Tidal volume (ml/kg predicted BW)	7 (7, 8)	7 (7, 8)	7 (7, 8)	0.835
FiO2 (%)	35 (30, 40)	35 (30, 40)	35 (30, 40)	0.101
PaO2 (mmHg)	71 (56, 92.5)	68.5 (55.9, 87.5)	72 (56.2, 95.8)	0.342
PaCO2 (mmHg)	45 (42, 47.8)	44.4 (42, 47.7)	45 (42, 48)	0.548
**Circulation parameters**				
Central venous pressure (mmHg)	8 (7, 10)	8 (7, 10)	9 (7, 10)	0.112
Lactate (mmol/L)	1.9 (1.3, 3, 3)	1.6 (1.2, 3.1)	2.1 (1.4, 3.4)	0.038
ScvO2 (%)	73 (68, 78)	71.5 (66.9, 78)	73 (69, 78)	0.036
cTnI (ug/L)	4.78 (1.55, 12.3)	2.9 (0.72, 9.23)	6.9 (2.8, 14.8)	<0.001
Nt-ProBNP (pg/ml)	1931 (956, 4931)	2115 (637.5, 6452.8)	1915 (1136, 4368)	0.727
LVEF (%)	53 (48, 58)	54 (48, 57)	53 (49, 58)	0.720
**Treatment on the first day in ICU**				
Prophylactic antibiotics (n/%)	327 (91.3%)	145 (94.2%)	182 (89.2%)	0.100
Acid-suppressing drugs	91 (25.4%)	41 (26.6%)	50 (24.5%)	0.649
Norepinephrine (n/%)	294 (82.1%)	129 (83.8%)	165 (80.9%)	0.481
Cardiotonic^#^ (n/%)	183 (51.1%)	83 (53.9%)	100 (49%)	0.361
CRRT (n/%)	59 (16.5%)	27 (17.5%)	32 (15.7%)	0.373
IABP (n/%)	14 (3.9%)	4 (2.6%)	10 (4.9%)	0.265
ECMO (n/%)	15 (4.2%)	5 (3.2%)	10 (4.9%)	0.439
**Laboratory evaluation**				
White blood cell (109/L)	12.7 (9.2, 16.3)	12.9 (9.3, 17.0)	12.6 (9.2, 16.2)	0.666
Neutrophil (109/L)	10.9 (7.9, 14.5)	10.9 (7.7, 15.1)	11.0 (7.9, 14.3)	0.925
Monocyte (109/L)	0.68 (0.45, 0.96)	0.66 (0.42, 1.01)	0.71 (0.47, 0.95)	0.438
Platelet (109/L)	125 (91, 164)	124.5 (89.5, 169)	125 (93, 164)	0.879
Creatinine (umol/L)	96.5 (77, 139)	103 (82.8, 151)	95 (67.3, 137)	0.009
Total bilirubin (umol/L)	17.7 (13.4, 27.9)	18.2 (14, 26.6)	16.9 (12.7, 28.3)	0.334
Albumin (g/L)	33 (31, 35)	33 (31, 35)	33 (31, 35)	0.929
Prothrombin Time (s)	14.1 (13.4, 15.4)	14.3 (13.4, 15.8)	14 (13.4, 15.1)	0.216
APTT-R	1.15 (1.01, 1.31)	1.14 (1.01, 1.35)	1.2 (1.0, 1.3)	0.268
Procalcitonin (ng/ml)	3.49 (1.0, 11.0)	4 (1.01, 13)	3.3 (0.98, 9.1)	0.218
hsCRP (mg/L)	142.9 (71.5, 239)	110.3 (49.8, 209.8)	167.1 (79, 256.4)	<0.0001
Interleukin-6 (pg/ml)	156 (54.3, 433)	121 (45.8, 364)	162 (69.6, 452)	0.146
Interleukin-8 (pg/ml)	102 (53.8, 171)	114 (55.5, 206)	91 (53, 155)	0.026
TNF-a (pg/ml)	12.6 (9.0, 16.8)	14.9 (10.9, 20.2)	11.2 (8.5, 15.3)	<0.0001

*PEEP, positive end expiratory pressure; FiO2, fraction of inspirated oxygen; PaO2, partial pressure of oxygen; PaCO2, partial pressure of carbon deoxidate; ScvO2, oxygen saturation of central venous blood; cTnI, cardiac troponin I; Nt-ProBNP, N-terminal pro brain natriuretic peptide; LVEF, left ventricular ejection fraction; CRRT, continuous renal replacement therapy; IABP, intra-aortic balloon pump; ECMO, extracorporeal membrane oxygenation; APTT-R, activated partial thromboplastin time ratio; hsCRP, hypersensitive C-reactive protein; TNF-a, tumor necrosis factor-a;*

# *Cardiotonics include epinephrine, dopamine, dobutamine, milrinone, levosimendan et al*.

The intensified lung physiotherapy bundle was well implemented and the rates of delirium assessment, early activity, enhanced airway drainage, recruitment maneuver, and high lateral position were significantly higher than that in the traditional bundle. When early mobilization or prone position is performed, the related complications are of great concern. The rate of endotracheal tube displacement was roughly the same as in the control group because the tubes were double fixed routinely in both the groups and there was no statistical difference in the incidence of postoperative bleeding and incision infection between the two groups (1.3 vs. 1.5% [*P* = 0.891] and 1.3 vs 2.0% [*P* = 0.629], respectively) because of the sternum reinforcement with steel plate lining during the operation and the external fixation with chest strap during early activity. We also routinely monitored the gastral residual volume every 6 h, and there was no significant difference in the rate of gastroesophageal reflux between the two groups. Compared with the control group, patients in the study group had a significantly lower rate of postoperative pneumonia (14.2 vs. 22.7%, *P* = 0.037), lower CPIS score (3 vs. 4, *P* = 0.01), and shorter mechanical ventilation time (92 vs. 144 h, *P* < 0.0001). The ICU and hospital stays were both statistically shorter in the study group than in the control group (5 vs. 7 days [*P* < 0.001] and 17 vs. 18.5 days [*P* = 0.022], respectively). The in-hospital mortality was also significantly lower in the study group (1.5 vs. 5.2%, *P* = 0.043) (Supplementary Table 1).

### Comparison of Postoperative Pneumonia and Non-pneumonia Patients

The incidence of postoperative pneumonia after high-risk cardiac surgery was 17.9% (64/358). To determine whether intensified treatment can reduce the incidence of postoperative pneumonia, we divided these patients into pneumonia (*n* = 64, 17.9%) and non-pneumonia (*n* = 294, 82.1%) groups.

The APACHE II and SOFA scores were higher in patients with postoperative pneumonia. Compared with the non-pneumonia patients, the proportion of emergency surgery, infective endocarditis, and dissection/aneurysm were much higher (in total 80.7 vs. 38.4%, *P* < 0.0001) in patients with pneumonia, and these patients received a higher rate of ECMO support (15.6 vs. 1.7%, *P* < 0.001). Patients with postoperative pneumonia had a higher cTnI level (7.8 vs. 4.6 μg L^−1^, *P* = 0.036) and lower left ventricular ejection fraction (LVEF) (48 vs. 55%, *P* < 0.0001). The level of IL-6 in patients with pneumonia was significantly lower than that in non-pneumonia patients (87 vs. 162 pg mL^−1^, *P* = 0.043) whereas the level of IL-8 was higher (131 vs. 97 pg mL^−1^, *P* = 0.026). The proportion of patients receiving the intensified lung physiotherapy bundle in the pneumonia group was much lower than that in the non-pneumonia group (14.2 vs. 59.5%, *P* = 0.037). The median CPIS score in the pneumonia patients was much higher (6 vs 3, *P* < 0.0001) and needed higher FiO_2_ (40 vs. 35%, *P* = 0.001). Patients with postoperative pneumonia had a longer duration of mechanical ventilation (279 vs. 96 h, *P* < 0.0001), ICU stay (13 vs. 5 days, *P* < 0.0001), and hospital stay (28.5 vs. 17 days, *P* < 0.0001), and the in-hospital mortality of these patients was also statistically higher (9.4 vs. 1.7%, *P* = 0.001) (Supplementary Table 2).

Multivariate logistic regression analysis was performed using factors with significant differences between the two groups in univariate analysis and factors of great value for the occurrence of postoperative pneumonia, such as prophylactic antibiotic use. It showed that surgery type (emergency or elective), ECMO application, and postoperative LVEF reduction were independent risk factors for postoperative pneumonia whilst the intensified lung physiotherapy bundle was a protective factor, which reduced the incidence of pneumonia (hazard ratio [HR] 2.717, 95% confidence interval 1.308–5.645, *P* = 0.007) (Table 3). Regarding the impact of postoperative pneumonia on in-hospital mortality, the incidence of postoperative pneumonia was much higher in the non-survivors (54.5 vs. 16.7%, *P* = 0.001). The intensified lung physiotherapy bundle was a predictor of in-hospital mortality based on the univariate analysis (27.3 vs. 57.9%, *P* = 0.043) (Supplementary Table 3) but was not significant on the multivariate analysis (adjusted HR 6.15, *P* = 0.172) (Supplementary Table 4).

**Table 3:** Multivariate logistic regression analysis of the factors affecting the occurrence of post-operative pneumonia.

	**B**	**Exp (B)**	**95%CI**		***P***
Intensified lung physiotherapy Bundle	0.930	2.534	1.267	5.067	0.009
COPD	−0.915	0.400	0.144	1.110	0.078
APACHE II	0.051	1.052	0.992	1.115	0.088
Surgery type (elective/emergency)	1.671	5.317	2.521	11.215	<0.0001
ECMO	−2.523	0.080	0.021	0.306	<0.0001
cTnI (pg/ml)	0.016	1.017	0.998	1.036	0.088
LVEF (%)	−0.083	0.920	0.883	0.960	<0.0001
Albumin (g/L)	−0.028	0.972	0.896	1.055	0.500
Prophylactic antibiotics	−0.834	0.434	0.114	1.656	0.222
Constant	0.467	0.627			0.810

*COPD, chronic obstructive pulmonary disease; APACHE II, acute physiology and chronic health evaluation; ECMO, extracorporeal membrane oxygenation; cTnI, cardiac troponin I; LVEF, left ventricular ejection fraction*.

## Discussion

To the best of our knowledge, this study is the first to explicitly elaborate on the role of an intensified lung physiotherapy bundle in cardiac surgery. The bundle potentially reduces the incidence of postoperative pneumonia in patients after cardiac surgery and significantly shortens the duration of mechanical ventilation, ICU stay, and hospital stay. Moreover, it is safe to implement, thus avoiding increased postoperative bleeding, incision infection, and other complications. The univariate analysis also showed that there may be a favorable impact on the prognosis of patients.

Pulmonary complications can be very common after cardiac surgery, and our study found that the incidence of pneumonia after high-risk cardiac surgery was as high as 17.9%, among which emergency surgery, infective endocarditis, and aneurysm accounted for a higher proportion, which was consistent with Kogan's study (1). Pulmonary complications after cardiac surgery are associated with high mortality (1, 10), and our study confirmed that the in-hospital mortality of patients with pneumonia was significantly increased in comparison with non-pneumonia patients (9.4 vs. 1.7%). Lung physiotherapy was necessary for post-cardiac surgery patients, although there was always a concern about its impact on bleeding and wound healing. We found that there was no significant difference in the incidence of postoperative bleeding, wound rupture or infection, and other complications between the two groups. Standardized lung physiotherapy after cardiac surgery is safe and is conducive to improving the prognosis of these patients (11).

As illustrated in previous literature (1, 12), we found that the incidence of postoperative pneumonia was related to the type of operation and the postoperative cardiac function, as the patients with pneumonia had mainly undergone emergency surgery, and their cTnI level and LVEF significantly differed from those of non-pneumonia patients. In our study, however, as a result of the intensified lung physiotherapy bundle, patients who had a higher cTnI level and lower LVEF had a lower incidence of postoperative pneumonia compared with the control group. This indicated that intensified lung physiotherapy was especially important in preventing postoperative pneumonia in patients with poor cardiac function after cardiac surgery. We are also aware that cardiac dysfunction can lead to pulmonary oedema and that emergency complex operations need longer CPB time, leading to a much fiercer inflammatory response and a more obvious impact on the patients. In this study, compared with the control group, the patients in the study group had much higher hsCRP and lower IL-8 levels, which equates to a stronger pro-inflammatory response and weaker anti-inflammatory response. To our surprise, under comparable prophylactic antibiotic administration and ventilator conditions, the patients in the study group had a statistically lower rate of postoperative pneumonia, further highlighting the importance of intensified lung physiotherapy in the setting of postoperative pneumonia.

This intensified bundle can reduce the incidence of postoperative pneumonia because it is designed to achieve physiological airway maintenance, while the provision of treatment closest to the patient's specific needs can realize better physiotherapeutic results. Compared with traditional lung physiotherapy, the intensified bundle has several advantages: first, use of the RASS to assess the depth of sedation and the CAM-ICU to assess delirium and facilitate early mobilization; second, use of the modified BOAS and Mucosal Plaque Score to assess oral care, and clear stipulation of the frequency and intensity of each intervention; third, adjustment of the degree of humidification in accordance with sputum characteristics; fourth, early use of auscultation, postural drainage, and vibration to facilitate airway clearance. In addition, whereas this bundle is able to significantly reduce the duration of mechanical ventilation, prolonged mechanical ventilation might result in an increased incidence of ventilator-associated pneumonia, resulting in a worse clinical outcome (13).

The intensified lung physiotherapy bundle was derived from the ventilator-associated pneumonia prevention bundle. As illustrated by many guidelines in many countries, the bundle should be implemented in accordance with the actual situation on the ward (14, 15). However, until now there have been no guidelines that clearly provide guidance or a flowchart regarding the procedures for lung physiotherapy. Our bundle was formulated in line with the actual clinical situation of our department and elicited from more than 20 years of clinical experience, with clear items and strong operability, while operational goals were set for each step of the protocol to make the process more feasible. Formulating the bundle was just the first step, with how to ensure effective implementation being a more important and difficult clinical challenge. In this intensified bundle, we tried to set operational goals using scales (such as RASS, CAM-ICU, and mBOAS) or a specific target for each measure to make the implementation more purposeful. For example, we provided clear provisions for the angle of patients when the lateral position was used to treat lung consolidation (such as left side 90°, right prone position 135°), and special protective measures were taken for these patients. Most importantly, we set up a special quality control group to supervise the implementation of various measures, from which we can learn and improve the bundle. It is gratifying to see that our bundle realistically reduced the incidence of postoperative pneumonia and shortened the duration of mechanical ventilation and hospital stay, and might also have benefits for in-hospital mortality. With detailed improvement and specific measures, as well as complete data and few cases lost to follow-up, our study can provide an effective reference guideline for lung physiotherapy in all kinds of patients.

Our study does have several limitations. First, on the comparison of the patients' characteristics and data from a large sample, the conclusion was persuasive. However, it was a controlled before-after study and, although the bias was conservative and few cases were lost to follow-up, more strict prospective randomized controlled trials are needed to further investigate the role of the intensified bundle on the postoperative pneumonia rate and duration of mechanical ventilation. Second, the relationship between covariates and outcome might be interactive or non-linear, the confounding factors need to be adjusted using ensemble modeling which can address non-linearity automatically in the future study (16). A multi-center study is needed to further refine the details of the process and verify the wide applicability of this intensified lung physiotherapy bundle.

## Conclusions

Our intensified lung physiotherapy bundle potentially reduces the rate of postoperative pneumonia after cardiac surgery and shortens the duration of mechanical ventilation. This bundle might also be adopted as a suitable reference guide for the prevention of other postoperative pulmonary complications.

## Data Availability Statement

The original contributions presented in the study are included in the article/Supplementary Material, further inquiries can be directed to the corresponding authors.

## Ethics Statement

This study was approved by the Institutional Review Board of PUMCH (approval number: JS-1170). Consent forms were obtained from patients' next of kin at ICU admission.

## Author Contributions

WC, HW, ZL, and NC contributed to the conception of the study, data interpretation, and drafting the manuscript. JC and JS contributed to the data collection and data analysis. JZ and DL contributed to data collection, interpretation, and critically revised the manuscript for important intellectual content. All authors approved the final version of the manuscript.

## Funding

This work was supported by the National Natural Science Foundation of China (No. 82072226), Beijing Municipal Science and Technology Commission (No. Z201100005520049), Non-profit Central Research Institute Fund of Chinese Academy of Medical Sciences (No. 2019XK320040), Tibet Natural Science Foundation [No. XZ2019ZR-ZY12(Z)], and Excellence Program of Key Clinical Specialty of Beijing in 2020 (No. ZK128001).

## Conflict of Interest

The authors declare that the research was conducted in the absence of any commercial or financial relationships that could be construed as a potential conflict of interest.

## Publisher's Note

All claims expressed in this article are solely those of the authors and do not necessarily represent those of their affiliated organizations, or those of the publisher, the editors and the reviewers. Any product that may be evaluated in this article, or claim that may be made by its manufacturer, is not guaranteed or endorsed by the publisher.

## Abbreviations

ICU: intensive care unit
RASS: richmond agitation sedation score
CAM-ICU: the confusion assessment method for ICU
BOAS: the modified beck oral assessment scale score
VIS: vasoactive-inotropic score
WBC: white blood cell
PEEP: positive end-expiratory pressure
FiO2: fraction of inspired oxygen
CRRT: continuous renal replacement therapy
IABP: intra-aortic balloon pump
ECMO: extracorporeal membrane oxygenation
SOFA: sequential organ failure assessment score
APACHE II: acute physiology and chronic health evaluation II score
CPIS: clinical pulmonary infection score
SD: standard deviation
CPB: cardiopulmonary bypass
cTnI: cardiac troponin I
IL: interleukin
LVEF: left ventricular ejection fraction
HR: hazard ratio.

## References

[1] Kogan A Segel MJ Ram E Raanani E Peled-Potashnik Y Levin S. Acute respiratory distress syndrome following cardiac surgery: comparison of the american-european consensus conference definition versus the berlin definition. Respiration. (2019) 97:518–24. 10.1159/000495511 30650409

[2] Stephens RS Shah AS Whitman GJ. Lung injury and acute respiratory distress syndrome after cardiac surgery. Ann Thorac Surg. (2013) 95:1122–9. 10.1016/j.athoracsur.2012.10.024 23352419

[3] Al-Qubati FAA Damag A Noman T. Incidence and outcome of pulmonary complications after open cardiac surgery, Thowra Hospital, Cardiac center, Sana'a, Yemen. Egypt J Chest Dis Tuberc. (2013) 62:775–80. 10.1016/j.ejcdt.2013.08.008

[4] Strickland SL Rubin BK Drescher GS Haas CF O'Malley CA Volsko TA. American Association for Respiratory Care IT: AARC clinical practice guideline: effectiveness of nonpharmacologic airway clearance therapies in hospitalized patients. Respir Care. (2013) 58:2187–93. 10.4187/respcare.02925 24222709

[5] Abdullahi A. Safety and efficacy of chest physiotherapy in patients with COVID-19: a critical review. Front Med (Lausanne). (2020) 7:454. 10.3389/fmed.2020.00454 32793618 PMC7385182

[6] Garcia-Delgado M Navarrete-Sanchez I Colmenero M. Preventing and managing perioperative pulmonary complications following cardiac surgery. Curr Opin Anaesthesiol. (2014) 27:146–52. 10.1097/ACO.0000000000000059 24514031

[7] Sun J Han W Cui N Li Q Wang H Li Z. Effect of nurse-led goal-directed lung physical therapy on the prognosis of pneumonia in sepsis patients in the ICU: a prospective cohort study. J Intensive Care Med. (2021) 37:258–66. 10.1177/0885066620987200 33511893

[8] Chen J Zhou R Li Z Li Q Long Y Wang H. Effect of nurse-led, goal-directed lung physiotherapy on prognosis of patients with sepsis caused by Acinetobacter baumannii pulmonary infection. Int J Infect Dis. (2021) 103:167–72. 10.1016/j.ijid.2020.11.196 33278626

[9] Gaies MG Gurney JG Yen AH Napoli ML Gajarski RJ Ohye RG. Vasoactive-inotropic score as a predictor of morbidity and mortality in infants after cardiopulmonary bypass. Pediatr Crit Care Med. (2010) 11:234–7. 10.1097/PCC.0b013e3181b806fc 19794327

[10] Piquilloud L. ARDS after cardiac surgery: is it a problem, a problem of definition, or both? Respiration. (2019) 97:495–7. 10.1159/000498948 31067542

[11] Martinsson A Houltz E Wallinder A Lindgren S Thoren A. Lung recruitment in the prone position after cardiac surgery: a randomised controlled study. Br J Anaesth. (2021) 126:1067–74. 10.1016/j.bja.2020.12.039 33602580

[12] Kor DJ Lingineni RK Gajic O Park PK Blum JM Hou PC. Predicting risk of postoperative lung injury in high-risk surgical patients: a multicenter cohort study. Anesthesiology. (2014) 120:1168–81. 10.1097/ALN.0000000000000216 24755786 PMC3999474

[13] Llitjos JF Reignier J Lascarrou JB. Prevention of early ventilator-associated pneumonia. N Engl J Med. (2020) 382:1672–3. 10.1056/NEJMx200010 32320588

[14] Kalil AC Metersky ML Klompas M Muscedere J Sweeney DA Palmer LB. Management of Adults With Hospital-acquired and Ventilator-associated Pneumonia: 2016 Clinical Practice Guidelines by the Infectious Diseases Society of America and the American Thoracic Society. Clin Infect Dis. (2016) 63:e61–e111. 10.1093/cid/ciw353 27418577 PMC4981759

[15] Zilberberg MD SA Kollef MH. Implementing quality improvements in the intensive care unit: ventilator bundle as an example. Crit Care Med. (2009) 37:305–9. 10.1097/CCM.0b013e3181926623 19050626

[16] Zhang Z Chen L Xu P Hong Y. Predictive analytics with ensemble modeling in laparoscopic surgery: a technical note. Laparosc Endosc Robot Surg. (2022). 10.1016/j.lers.2021.12.003 (in Press).

