# Supplementary material for: Role of Intensified Lung Physiotherapy Bundle on the Occurrence of Pneumonia After Cardiac Surgery
Source: Front Med (Lausanne). 2022 Feb 23;9:844094. doi: 10.3389/fmed.2022.844094 (PMC8904720; doi:10.3389/fmed.2022.844094)
Supplement: Supplementary file 1 [file Data_Sheet_1.docx]

**Supplement Materials**

**Supplement Table 1**

Clinical characteristics of cardiac surgery patients at ICU admission

**Supplement Table 2**

Comparison of bundle performance and prognosis-related parameters between study and control group

**Supplement Table 3**

Comparison of post-operative pneumonia and non-pneumonia patients

**Supplement Table 4**

Comparison between non-survivors and survivors according to in-hospital mortality

**Supplement Table 5**

Multivariate analysis of factors affecting in-hospital mortality

**Supplement Table 1** clinical characteristics of cardiac surgery patients at ICU admission

|  | Total | Control group | Study group | P |
| --- | --- | --- | --- | --- |
| **Vtial signs at admission** |  |  |  |  |
| Heart rate (beats per minute) | 104 (92,113) | 103 (92,111) | 104 (93,115) | 0.197 |
| Mean artery pressure (mmHg) | 82 (75,88) | 85 (75,88) | 81 (75,88) | 0.178 |
| Temperature (℃) | 37.8 (37.3,38.2) | 37.8 (37.3,38.1) | 37.8 (37.3,38.2) | 0.314 |
| Respiratory rate (per minute) | 16 (15,20) | 17 (15,23) | 16 (15,18) | 0.01 |
| **Ventilation parameters** |  |  |  |  |
| PEEP (cmH2O) | 6（5，8） | 6（5，8） | 6（5.8） | 0.347 |
| Tidal volume (ml/kg predicted BW) | 7（7，8） | 7（7，8） | 7（7，8） | 0.835 |
| FiO2 (%) | 35 (30,40) | 35 (30,40) | 35 (30,40) | 0.101 |
| PaO2 (mmHg) | 71 (56,92.5) | 68.5 (55.9,87.5) | 72 (56.2,95.8) | 0.342 |
| PaCO2 (mmHg) | 45 (42,47.8) | 44.4 (42,47.7) | 45 (42,48) | 0.548 |
| **Circulation parameters** |  |  |  |  |
| Central venous pressure (mmHg) | 8 (7,10) | 8 (7,10) | 9 (7,10) | 0.112 |
| Lactate (mmol/L) | 1.9 (1.3,3,3) | 1.6 (1.2,3.1) | 2.1 (1.4,3.4) | 0.038 |
| ScvO2 (%) | 73 (68,78) | 71.5 (66.9,78) | 73 (69,78) | 0.036 |
| cTnI (ug/L) | 4.78 (1.55,12.3) | 2.9 (0.72,9.23) | 6.9 (2.8,14.8) | <0.001 |
| Nt-ProBNP (pg/ml) | 1931 (956,4931) | 2115 (637.5,6452.8) | 1915 (1136,4368) | 0.727 |
| LVEF (%) | 53 (48,58) | 54 (48,57) | 53 (49,58) | 0.720 |
| **Treatment on the first day in ICU** |  |  |  |  |
| Prophylactic Antibiotics (n/%) | 327（91.3%） | 145（94.2%） | 182（89.2%） | 0.100 |
| Norepinephrine (n/%) | 294（82.1%） | 129（83.8%） | 165（80.9%） | 0.481 |
| Cardiotonic^#^ (n/%) | 183（51.1%） | 83（53.9%） | 100（49%） | 0.361 |
| CRRT (n/%) | 59（16.5%） | 27（17.5%） | 32（15.7%） | 0.373 |
| IABP (n/%) | 14（3.9%） | 4（2.6%） | 10（4.9%） | 0.265 |
| ECMO (n/%) | 15（4.2%） | 5（3.2%） | 10（4.9%） | 0.439 |
| **Laboratory evaluation** |  |  |  |  |
| White blood cell (*109/L) | 12.7 (9.2,16.3) | 12.9 (9.3,17.0) | 12.6 (9.2,16.2) | 0.666 |
| Neutrophil (*109/L) | 10.9 (7.9,14.5) | 10.9 (7.7,15.1) | 11.0 (7.9,14.3) | 0.925 |
| Monocyte (*109/L) | 0.68 (0.45,0.96) | 0.66 (0.42,1.01) | 0.71 (0.47,0.95) | 0.438 |
| Platelet (*109/L) | 125 (91,164) | 124.5 (89.5,169) | 125 (93,164) | 0.879 |
| Creatinine (umol/L) | 96.5 (77,139) | 103 (82.8,151) | 95 (67.3,137) | 0.009 |
| Total Bilirubin (umol/L) | 17.7 (13.4,27.9) | 18.2 (14,26.6) | 16.9 (12.7,28.3) | 0.334 |
| Albumin (g/L) | 33 (31,35) | 33 (31,35) | 33 (31,35) | 0.929 |
| Prothrombin Time (s) | 14.1 (13.4,15.4) | 14.3 (13.4,15.8) | 14 (13.4,15.1) | 0.216 |
| APTT-R | 1.15 (1.01,1.31) | 1.14 (1.01,1.35) | 1.2 (1.0,1.3) | 0.268 |
| Procalcitonin (ng/ml) | 3.49 (1.0,11.0) | 4 (1.01,13) | 3.3 (0.98,9.1) | 0.218 |
| hsCRP (mg/L) | 142.9 (71.5,239) | 110.3 (49.8,209.8) | 167.1 (79,256.4) | <0.0001 |
| Interleukin-6 (pg/ml) | 156 (54.3,433) | 121 (45.8,364) | 162 (69.6,452) | 0.146 |
| Interleukin-8 (pg/ml) | 102 (53.8,171) | 114 (55.5,206) | 91 (53,155) | 0.026 |
| TNF-a (pg/ml) | 12.6 (9.0,16.8) | 14.9 (10.9,20.2) | 11.2 (8.5,15.3) | <0.0001 |

PEEP positive end expiratory pressure; FiO2 fraction of inspirated oxygen; PaO2 partial pressure of oxygen; PaCO2 partial pressure of carbon deoxidate; ScvO2 oxygen saturation of central venous blood; cTnI Cardiac troponin I; Nt-ProBNP N-terminal pro brain natriuretic peptide; LVEF left ventricular ejection fraction; CRRT continuous renal replacement therapy; IABP intra-aortic balloon pump; ECMO extracorporeal membrane oxygenation; APTT-R activated partial thromboplastin time ratio; hsCRP hypersensitive C-reactive protein; TNF-a tumor necrosis factor-a.

**Supplement Table 2** Comparison of bundle performance and prognosis-related parameters between study and control group

|  | Total | Control group  N=154 | Study group  N=204 | P |
| --- | --- | --- | --- | --- |
| **Bundle performance** |  |  |  |  |
| Delirium assessment | 217(60.6%) | 13(8.4%) | 204(100%) | <0.0001 |
| Early activity  Bed activity  Bedside activity | 198(55.3%)  94(26.3%) | 37(24%)  11(7.1%) | 161(78.9%)  83(40.7%) | <0.0001  <0.0001 |
| Enhanced airway drainage | 228(63.7%) | 24(15.6%) | 204(100%) | <0.0001 |
| Recruitment maneuver | 182(50.8%) | 7(4.5%) | 175(85.8%) | <0.0001 |
| High lateral position | 195(54.5%) | 9(5.8%) | 186(91.2%) | <0.0001 |
| **Prognosis related parameters** |  |  |  |  |
| Post-operative bleeding | 5 (1.4%) | 2 (1.3%) | 3 (1.5%) | 0.891 |
| Incision infection | 6 (1.7%) | 2 (1.3%) | 4 (2.0%) | 0.629 |
| Post-operative pneumonia | 64 (17.9%) | 35 (22.7%) | 29 (14.2%) | 0.037 |
| CPIS score | 3 (3,4) | 4 (3,5.75) | 3 (3,4) | 0.01 |
| Mechanical ventilation time (h) | 107 (72，167.25) | 144 (96，189.25) | 92 (55，140.75) | <0.0001 |
| Hospital stay (days) | 18 (14，18) | 18.5 (14，27) | 17 (13，25) | 0.022 |
| ICU stay (days) | 6 (4，6) | 7 (5，7) | 5 (4，7) | <0.001 |
| In-hospital mortality | 11 (3.1%) | 8 (5.2%) | 3 (1.5%) | 0.043 |

CPIS clinical pulmonary infection score; ICU intensive care unit.

**Supplement Table 3** Comparison of post-operative pneumonia and non-pneumonia patients

|  | Total | Pneumonia（64） | Non-pneumonia（294） | P |
| --- | --- | --- | --- | --- |
| Age (years) | 59 (47，67) | 61(48,68) | 58 (46,66) | 0.289 |
| Sex（male） | 238 (66.5) | 38 (59.4) | 200 (68%) | 0.184 |
| Underlying disease |  |  |  |  |
| Hypertension | 103 (28.8%) | 37.5% | 26.9% | 0.089 |
| Diabetes Miletus | 48 (13.4%) | 5 (7.8%) | 43 (14.6%) | 0.147 |
| COPD | 28 (7.8%) | 10 (15.6%) | 18 (6.1%) | 0.010 |
| Chronic kidney disease | 13 (3.6%) | 3 (4.7%) | 10 (3.4%) | 0.618 |
| Chronic liver disease | 13 (3.6%) | 3 (4.7%) | 10 (3.4%) | 0.618 |
| Apache II | 16 (13，20) | 17.5 (15,23) | 16 (12,19.8) | 0.002 |
| SOFA | 10 (8，12) | 11 (9.25,13) | 10 (8,12) | 0.011 |
| **Operation-related parameters** |  |  |  |  |
| Operation type |  |  |  | <0.0001 |
| Emergency surgery | 71 (19.8%) | 28 (43.8%) | 43 (14.6%) |  |
| Pericardial surgery | 21 (5.9%) | 2 (3.1%) | 19 (6.5%) |  |
| Dissection/aneurysm | 37 (10.3%) | 12 (18.8%) | 25 (8.5%) |  |
| Infectious endocarditis | 57 (15.9%) | 12 (18.8%) | 45(15.3%) |  |
| Elective CABG/Valve surgery | 132 (36.9%) | 5 (7.8%) | 127 (43.2%) |  |
| Others* | 39 (10.9%) | 5 (7.8%) | 34 (11.6%) |  |
| Cardiopulmonary bypass time (min) | 128.5 (96，173.5) | 147 (109.25, 176.75) | 124 (93,172) | 0.314 |
| Aortic cross clamp duration (min) | 87 (56，119) | 90.5 (70.25,116.5) | 87 (54,119) | 0.062 |
| **Vital signs at ICU admission** |  |  |  |  |
| Heart rate (beats per minute) | 104 (92,113) | 100 (90,111) | 104 (93,114) | 0.279 |
| Mean artery pressure (mmHg) | 82 (75,88) | 82 (75,87) | 82.5 (75,88) | 0.636 |
| Temperature (℃) | 37.8 (37.3,38.2) | 37.9 (27,38.4) | 37.8 (37.4,38.2) | 0.532 |
| Respiratory rate (per minute) | 16 (15,20) | 16 (15,21) | 16 (15,19) | 0.791 |
| **Ventilation parameters** |  |  |  |  |
| PEEP (cmH2O) | 6 (5，8) | 6 (5，8) | 6 (5，8) | 0.485 |
| Tidal volume (ml/kg predicted BW) | 7 (7，8) | 7 (7，7) | 7 (7，8) | 0.066 |
| FiO2 (%) | 35 (30,40) | 40 (35,45) | 35 (30,40) | 0.001 |
| PaO2 (mmHg) | 71 (56,92.5) | 68 (55.9,96) | 72 (56,92) | 0.846 |
| PaCO2 (mmHg) | 45 (42,47.8) | 45.6 (41.7, 48) | 44.8 (42,47.7) | 0.920 |
| **Circulation parameters** |  |  |  |  |
| Central venous pressure (mmHg) | 8 (7,10) | 8 (7,10) | 8 (7,10) | 0.750 |
| Lactate (mmol/L) | 1.9 (1.3,3,3) | 2.2 (1.4,4.0) | 1.8 (1.2,3.2) | 0.169 |
| ScvO2 (%) | 73 (68,78) | 73 (68.2, 80) | 73 (68,78) | 0.257 |
| cTnI (ug/L) | 4.78 (1.55,12.3) | 7.8 (2.27,16.3) | 4.6 (1.4,11.7) | 0.036 |
| Nt-ProBNP (pg/ml) | 1931 (956,4931) | 2382 (917, 8059) | 1920 (965,4679) | 0.471 |
| LVEF (%) | 53 (48,58) | 48 (43.2, 52.8) | 55 (50,58) | <0.0001 |
| **Treatment on the first day in ICU** |  |  |  |  |
| Prophylactic Antibiotics (n/%) | 327 (91.3%) | 59 (92.2%) | 268 (91.2%) | 0.790 |
| Norepinephrine (n/%) | 294 (82.1%) | 55 (85.9%) | 239 (81.3%) | 0.379 |
| Cardiotonic^#^ (n/%) | 183 (51.1%) | 35 (54.7%) | 148 (50.3%) | 0.528 |
| CRRT (n/%) | 59 (16.5%) | 14 (21.9%) | 50 (17%) | 0.357 |
| IABP (n/%) | 14 (3.9%) | 1 (1.6%) | 13 (4.4%) | 0.285 |
| ECMO (n/%) | 15 (4.2%) | 10 (15.6%) | 5 (1.7%) | <0.001 |
| **Laboratory evaluation** |  |  |  |  |
| White blood cell (*109/L) | 12.7 (9.2,16.3) | 12.7 (9.9,15.4) | 12.6 (9.2,16.7) | 0.579 |
| Neutrophil (*109/L) | 10.9 (7.9,14.5) | 10.9 (8.3,13.6) | 11.0 (7.7,14.7) | 0.583 |
| Monocyte (*109/L) | 0.68 (0.45,0.96) | 0.62 (0.42,0.89) | 0.69 (0.46,0.97) | 0.181 |
| Platelet (*109/L) | 125 (91,164) | 114 (87,162.8) | 127 (93.8,169) | 0.180 |
| Creatinine (umol/L) | 96.5 (77,139) | 110 (83,159.8) | 96 (74,137.5) | 0.077 |
| Total Bilirubin (umol/L) | 17.7 (13.4,27.9) | 18.6 (14.1,32.9) | 17.4 (13,26.1) | 0.174 |
| Albumin (g/L) | 33 (31,35) | 32 (30,34) | 33 (31,35) | 0.007 |
| Prothrombin Time (s) | 14.1 (13.4,15.4) | 13.7 (13.0,15.4) | 14.2 (13.4,15.4) | 0.072 |
| APTT-R | 1.15 (1.01,1.31) | 1.16 (1.01,1.35) | 1.14 (1.01,1.29) | 0.524 |
| Procalcitonin (ng/ml) | 3.49 (1.0,11.0) | 2.9 (0.98,11.5) | 3.5 (1,11) | 0.931 |
| hsCRP (mg/L) | 142.9 (71.5,239) | 160 (52.9,212) | 141 (71.8,246) | 0.354 |
| Interleukin-6 (pg/ml) | 156 (54.3,433) | 87 (37.8,310) | 162 (65.6,449.3) | 0.043 |
| Interleukin-8 (pg/ml) | 102 (53.8,171) | 131 (64,210) | 97 (53,161.5) | 0.026 |
| TNF-a (pg/ml) | 12.6 (9.0,16.8) | 13.8 (10.8,17.8) | 12.4 (8.88,16.5) | 0.115 |
| **Prognosis-related parameters** |  |  |  |  |
| Intensified physiotherapy Bundle (Yes) | 204 (56.9%) | 29 (14.2%) | 175 (59.5%) | 0.037 |
| Post-operative bleeding | 5 (1.4%) | 1 (1.6%) | 4 (1.4%) | 0.901 |
| Incision infection | 6 (1.7%) | 1 (1.6%) | 5 (1.7%) | 0.938 |
| CPIS score | 3 (3,4) | 5 (4,7,75) | 3.0 (3,4) | <0.0001 |
| Mechanical ventilation time (h) | 107 (72，167.25) | 279 (216, 338) | 96 (66.8,135) | <0.0001 |
| Hospital stays (days) | 18 (14，18) | 28.5 (21,40.75) | 17 (13,23) | <0.0001 |
| ICU stays (days) | 6 (4，6) | 13 (10,15.8) | 5 (4,7) | <0.0001 |
| In-hospital mortality | 11 (3.1%) | 6(9.4%) | 5(1.7%) | 0.001 |

*Other surgeries include Pulmonary endarterectomy and congenital heart disease surgery;

#Cardiotonics include epinephrine, dopamine, dobutamine, milrinone, levosimendan et al.

COPD chronic obstructive pulmonary disease; Apache II Acute Physiology and Chronic Health Evaluation II; SOFA sequential organ failure assessment；CABG Coronary artery bypass grafting; CRRT continuous renal replacement therapy; IABP intra-aortic balloon pump; ECMO extracorporeal membrane oxygenation; PEEP positive end expiratory pressure; FiO2 fraction of inspirated oxygen; PaO2 partial pressure of oxygen; PaCO2 partial pressure of carbon deoxidate; ScvO2 oxygen saturation of central venous blood; Pv-aCO2 Venous arterial partial pressure difference of carbon dioxide; cTnI Cardiac troponin I; Nt-ProBNP N-terminal pro brain natriuretic peptide; LVEF left ventricular ejection fraction; APTT-R activated partial thromboplastin time ratio; hsCRP hypersensitive C-reactive protein; TNF-a tumor necrosis factor-a; CPIS clinical pulmonary infection score.

**Supplement Table 4** Comparison between non-survivors and survivors according to in-hospital mortality

|  | Non-survivors(n=11) | Survivors(n=347) | P |
| --- | --- | --- | --- |
| Apache II | 18 (14,27) | 16 (13,20) | 0.012 |
| SOFA | 13 (11,16) | 10 (8,12) | <0.0001 |
| Lactate (mmol/L) | 3.2 (1.4,6.2) | 1.85 (1.3,3.2) | 0.001 |
| LVEF (%) | 46 (38,54) | 53 (49,58) | 0.036 |
| CPIS | 6 (3,9) | 3 (3,4) | 0.046 |
| Prothrombin time (s) | 14.4 (13.5,18.4) | 14.1 (13.4,15.3) | 0.01 |
| APTT-R | 1.3 (1.14,1.42) | 1.14 (1.01,1.30) | 0.012 |
| Interleukin-10 (pg/ml) | 20.5 (8,74.7) | 8.8 (5.3,16.5) | 0.019 |
| TNF-a (pg/ml) | 15.7 (11.1,19.6) | 12.5 (8.9,16.6) | 0.027 |
| Procalcitonin (ng/ml) | 7.56 (0.98,34.1) | 3.4 (1.0,10.8) | 0.038 |
| ECMO | 54.5% (6/11) | 2.6% (9/347) | <0.0001 |
| Post-operative Pneumonia | 54.5% (6/11) | 16.7% (58/347) | 0.001 |
| Intensified lung physiotherapy Bundle | 27.3% (3/11) | 57.9% (201/347) | 0.043 |

Apache II Acute Physiology and Chronic Health Evaluation; SOFA sequential organ failure assessment; LVEF left ventricular ejection fraction; CPIS clinical pulmonary infection score; APTT-R Activated partial thromboplastin time ratio; TNF-a tumor necrosis factor-a; ECMO extracorporeal membrane oxygenation.

**Supplement Table 5** Multivariate analysis of factors affecting in-hospital mortality

|  | B | exp（B） | 95%CI | | P |
| --- | --- | --- | --- | --- | --- |
| Intensified lung physiotherapy Bundle | 1.816 | 6.146 | 0.454 | 83.227 | 0.172 |
| Apache II | 0.069 | 1.071 | 0.829 | 1.384 | 0.599 |
| SOFA | 0.199 | 1.221 | 0.838 | 1.778 | 0.299 |
| Lactate | -0.132 | 0.876 | 0.537 | 1.429 | 0.596 |
| LVEF | -0.133 | 0.875 | 0.735 | 1.042 | 0.135 |
| CPIS | 0.579 | 1.784 | 0.876 | 3.634 | 0.111 |
| Interleukin-10 | 0.004 | 1.004 | 0.981 | 1.027 | 0.744 |
| TNF-a | -0.004 | 0.996 | 0.957 | 1.037 | 0.846 |
| Procalcitonin | 0.054 | 1.055 | 1.006 | 1.107 | 0.027 |
| ECMO | -2.749 | 0.064 | 0.002 | 2.298 | 0.132 |
| Post-operative pneumonia | 2.168 | 8.741 | 0.151 | 505.593 | 0.295 |
