# Supplementary figures and images for: Role of Intensified Lung Physiotherapy Bundle on the Occurrence of Pneumonia After Cardiac Surgery
Source: Front Med (Lausanne). 2022 Feb 23;9:844094. doi: 10.3389/fmed.2022.844094 (PMC8904720; doi:10.3389/fmed.2022.844094)

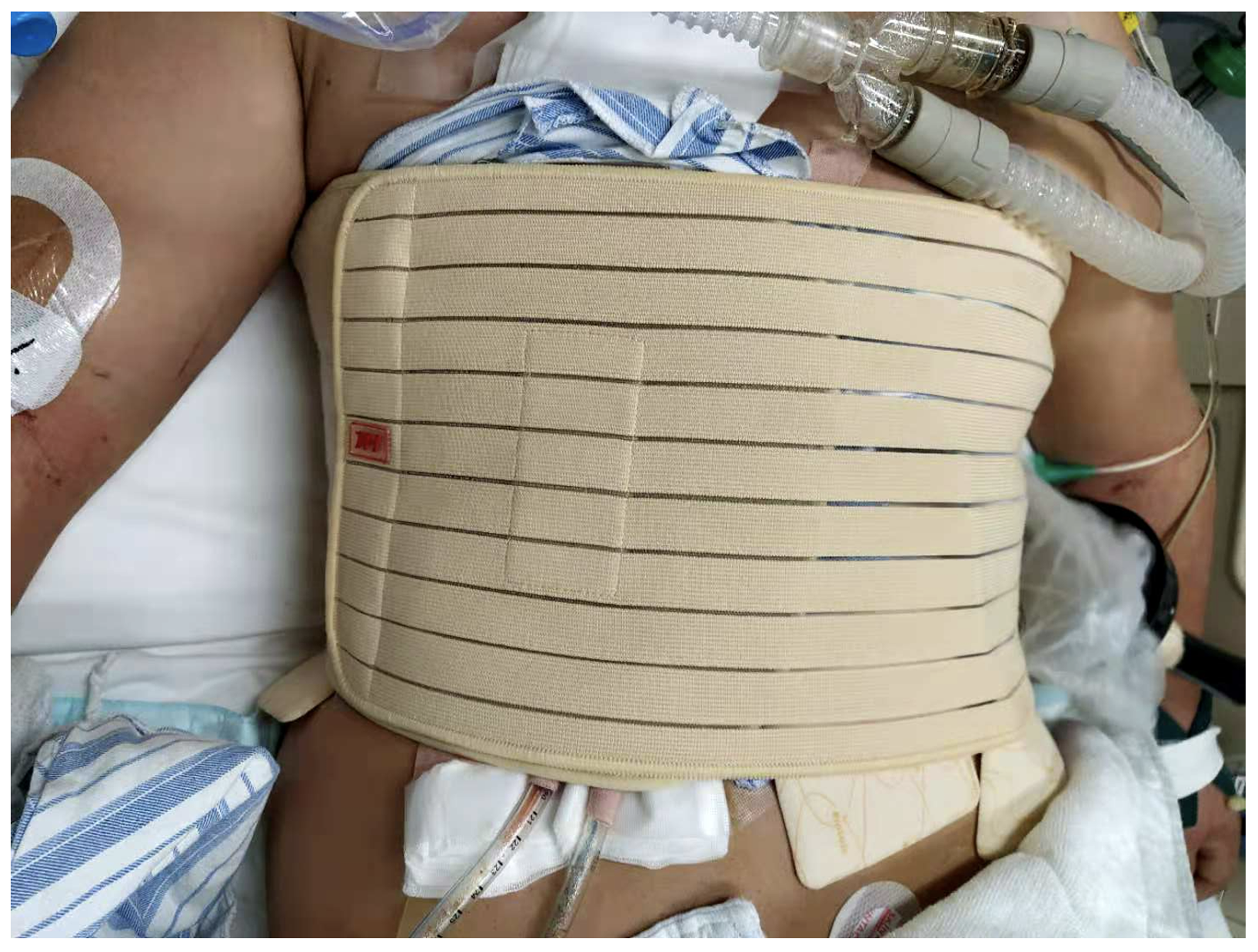

Supplement: Supplementary file 2 [file Image_1.PNG]
